# Emotion dysregulation mediates the relationship between nightmares and psychotic experiences: results from a student population

**DOI:** 10.1038/s41537-020-0103-y

**Published:** 2020-06-01

**Authors:** Umair Akram, Maria Gardani, Kamila Irvine, Sarah Allen, Antonia Ypsilanti, Lambros Lazuras, Jennifer Drabble, Jodie C. Stevenson, Asha Akram

**Affiliations:** 1 Centre for Behavioural Science and Applied Psychology, Sheffield Hallam University, Sheffield, UK; 2 Nuffield Department of Clinical Neurosciences, University of Oxford, Oxford, UK; 3 School of Psychology, University of Glasgow, Glasgow, UK; 4 School of Psychology, University of Lincoln, Lincoln, UK; 5 School of Social Sciences, University of Teesside, Middlesbrough, UK; 6 Department of Psychology, The University of Sheffield, Sheffield, UK

**Keywords:** Psychosis, Schizophrenia, Human behaviour

## Abstract

Sleep disruption is commonly associated with psychotic experiences. While sparse, the literature to date highlights nightmares and related distress as prominent risk factors for psychosis in students. We aimed to further explore the relationship between specific nightmare symptoms and psychotic experiences in university students while examining the mediating role of emotion dysregulation. A sample (*N* = 1273) of student respondents from UK universities completed measures of psychotic experiences, nightmare disorder symptomology and emotion dysregulation. Psychotic experiences were significantly more prevalent in students reporting nightmares (*n* = 757) relative to those who did not (*n* = 516). Hierarchical linear regression analysis showed that psychotic experiences were significantly associated (Adjusted *R*^2^ = 32.4%) with perceived nightmare intensity, consequences and resulting awakenings, and with emotion regulation difficulties. Furthermore, multiple mediation analysis showed that the association between psychotic experiences and nightmare factors was mediated by emotion regulation difficulties. Adaptive regulation of dream content during rapid eye-movement sleep has previously been demonstrated to attenuate surges in affective arousal by controlling the intensity and variability of emotional content. Difficulties in emotion regulation may partially explain the experience of more intense and disruptive nightmares among individuals with psychotic experiences. Emotion regulation may represent an important control mechanism that safeguards dream content and sleep quality.

## Introduction

Disturbed sleep plays a crucial role in predicting the development of first-onset psychosis and paranoid thinking^1,2^. Specifically, abnormalities in objective sleep continuity (i.e. increased sleep onset latency and fragmentation) and circadian rhythm disruption are commonly reported in people experiencing psychosis^2–4^. Sleep disruption at disorder level (i.e. insomnia and obstructive sleep apnoea) has also been evidenced in this population^4–6^. However, while persistent reports of childhood nightmares appear to predict future psychotic experiences in young adulthood^2^, the role of nightmares in relation to psychotic experiences in young adults has received little attention until recently^7,8^.

Nightmares are highly prevalent during adolescence and young adulthood^1,9^. In the general population, weekly nightmares present at a rate of up to 6%^10,11^. In contrast, approximately 19% of young students aged between 15 and 17 years frequently experience clinically severe nightmares^12,13^. Vivid, dysphoric, and followed by startling awakenings, nightmares entail frightening dreams composed of threats to survival, security or physical integrity resulting in significant daytime impairment and reduced quality of life^14,15^. Nightmares are commonly associated with poor physical health^16^, marked psychological distress^9^, and symptoms of anxiety, depression, post-traumatic stress and psychosis^16,17^. Sheaves et al.^7^ were the first to examine the occurrence of nightmares among patients with psychosis. With the use of a retrospective dream log, over half of the sample (55%) reported weekly distressing nightmares indicative of severe pathology—a rate markedly higher than in the general population (0.9– 6.8%)^11,14^. Only one study examined the relationship between nightmares and psychotic experiences in UK students as part of The Oxford Sleep Survey, and found that a dose–response increase in nightmare frequency and associated distress was positively associated with higher scores in psychiatric difficulty (i.e. hallucinations, paranoia, depression, anxiety and hypomania)^8^.

Exposed to significant life changes (e.g. increased independence and social demands) and academic challenges (e.g. independent learning) accompanied with the loss of parental support and oversight, university students are particularly vulnerable to psychological distress and the development or exacerbation of psychiatric difficulty^18^. Given emerging evidence that nightmares and their associated distress confer risk for psychosis in students^8,9^, it is important to empirically examine mediating variables that may be amenable to mental health interventions.

Emotion regulation refers to the ability to identify, understand and accept emotions; control impulsive behaviours in a way that aligns with one’s goals; and use appropriate strategies to moderate emotional reactivity^19^. Rather than the absence of regulation, emotion dysregulation refers to inflexible strategies that interfere with social, cognitive or interpersonal functioning^20^. Here, difficulties in four broad domains are proposed: awareness and understanding of emotions; acceptance of emotions; ability to control impulses and behave in accordance with goals in the presence of negative affect; and access to effective emotion regulation strategies^20^.

Theoretical models of nightmares identify emotion regulation as a vital function of dreaming. More specifically, nightmares are considered to emerge as either admonitory expressions or failed suppression of emotionally intense content that negatively influences one’s waking mood state the following day^21^. Emotion dysregulation is also regarded a key feature of psychosis, yet despite evidence of emotional difficulties in psychotic disorders, outcomes remain mixed. Nevertheless, research highlights that difficulties in the identification and acceptance of emotions to be characteristic of this population^21–24^. Therefore, we consider emotion dysregulation may possibly mediate the relationship between nightmares and psychotic experiences among students, potentially serving as a novel focal point for treatment.

In this paper, we further explore the relationship between nightmare symptoms and psychotic experiences in university students while examining the potential mediating role of emotion dysregulation. Specifically, we examined if psychotic experiences are greater among those reporting nightmares, and if emotion dysregulation would mediate the association between nightmare characteristics (i.e. severity, intensity, frequency, awakenings, perceived consequences) and psychotic experiences. In line with previous research^7–11^, we predicted nightmare characteristics to be positively associated with psychotic experiences. Next, while we generally expect some domains of emotion dysregulation to mediate these relationships, this latter aim is considered exploratory in nature with no directional hypothesis. This is due to mixed evidence concerning which specific difficulties in emotion regulation are observed among individuals presenting psychosis.

## Results

One-way analysis of variance (ANOVA) showed that psychotic experiences (4.84 ± 3.54) were significantly more prevalent in students reporting nightmares (and subsequently completing the DDNSI: *n* = 757) relative to those who did not (*n* = 516: 3.09 ± 2.83), *F*(1, 1255) = 86.96, *p* < 0.0001, Cohen’s *d* = 0.55. Mean scores for the final sample completing the DDNSI are presented in Table 1.

**Table 1:** Mean scores (± standard deviations) of psychotic experiences, nightmares and emotion dysregulation for the final sample completing the DDNSI (*N* = 757).

	Range	Mean score
Psychotic experiences	0–16	4.84 ± 3.38
*DDNSI*		
Composite	3–37	14.22 ± 5.37
Severity	0–6	2.40 ± 1.17
Intensity	0–6	2.95 ± 1.22
Frequency	2–14	4.67 ± 2.32
Awakenings	0–4	1.87 ± 1.17
Consequences	0–30	8.18 ± 6.24
*DERS-SF*		
Nonacceptance	3–15	8.44 ± 3.57
Goals	3–15	10.28 ± 3.39
Impulse	3–15	6.08 ± 3.36
Awareness	3–15	7.43 ± 2.95
Strategies	3–15	7.91 ± 3.45
Clarity	3–15	7.21 ± 3.02

Note: *DDNSI* Disturbing Dreams and Nightmare Severity Index, *DERS-SF* Difficulties in Emotion Regulation Scale Short Form.

### Correlations between nightmare symptoms and psychotic experiences

Among individuals reporting nightmares, increased psychotic experiences were positively associated with the severity (*r* = 0.22, *p* = 0.001), intensity (*r* = 0.25, *p* = 0.001), frequency (*r* = 0.21, *p* = 0.001), resulting awakenings (*r* = 0.08, *p* = 0.02) and perceived consequences (*r* = 0.42, *p* = 0.001) of reported nightmares. Likewise, total nightmare scores were also positively associated with psychotic experiences (*r* = 0.26, *p* = 0.001). The results from the correlation analysis are summarised in Table 2.

**Table 2:** Correlations between psychotic experiences, nightmares and emotion dysregulation.

	1.	2.	3.	4.	5.	6.	7.	8.	9.	10.	11.	12.
1. Psychotic experiences												
*DDNSI*												
2. Composite	0.26**											
3. *Consequences*	0.42**	0.55**										
4. Severity	0.22**	0.73**	0.53**									
5. Intensity	0.25**	0.71**	0.48**	0.67**								
6. Frequency	0.21**	0.87**	0.39**	0.42**	0.38**							
7. Awakenings	0.08*	0.54**	0.32**	0.40**	0.42**	0.21**						
*DERS-SF*												
80. *Nonacceptance*	0.42**	0.30**	0.42**	0.26**	0.22**	0.25**	0.14**					
90. Goals	0.31**	0.22**	0.33**	0.17**	0.19**	0.17**	0.12**	0.48**				
100. Impulse	0.42**	0.24**	0.36**	0.21**	0.15**	0.22**	0.07	0.48**	0.48**			
11. Awareness	0.17**	0.03	0.11**	0.01	−0.01	0.06	−0.04	0.11**	−0.06*	0.06*		
12. Strategies	0.47**	0.30**	0.44**	0.23**	0.25**	0.25**	0.12**	0.62**	0.62**	0.67**	0.11**	
13. Clarity	0.46**	0.20**	0.32**	0.17**	0.15**	0.18**	0.07*	0.49**	0.35**	0.43**	0.29**	0.53**

Note: Psychotic experiences, Prodromal-16 Count; *DDNSI* Disturbing Dreams & Nightmare Severity Index, *DERS-SF* Difficulties in Emotion Regulation Scale-SF. Correlations are shown for the final sample completing the DDNSI.

*p < 0.05, **p < 0.01.

### Direct association between nightmare symptoms, emotion dysregulation and psychotic experiences

A hierarchical multiple regression analysis was used to examine the association of nightmare symptoms and emotion dysregulation with psychotic experiences. The analysis was completed in two steps and the first step included nightmare symptoms (i.e., severity, intensity, frequency, resulting awakenings and perceived consequences of nightmares), and emotion dysregulation dimensions were added in the second step. An overall significant model, *F*(10, 743) = 36.5, *p* < 0.001, emerged predicting 32.4% (Adjusted *R*^2^) of the variance in psychotic experiences, and tolerance levels were acceptable (>0.361), thus, suggesting that the predictor variables were independently associated with the criterion variable. In the first step, psychotic experiences were significantly associated with nightmare intensity (*ß* = 0.11, *p* = 0.01), resulting awakenings (*ß* = −0.08, *p* = 0.02) and perceived consequences (*ß* = 0.41, *p* < 0.0001) of nightmares. In the second step of the analysis, the addition of emotion dysregulation dimensions significantly increased predicted variance by 14.8%, *F*_change_(6, 733) = 27.06, *p* < 0.001. Intensity and perceived consequences of nightmares retained their significant association with psychotic experiences, but the effect of resulting awakening turned marginally non-significant (*ß* = −0.06, *p* = 0.07). All the emotion dysregulation dimensions but goals were significantly associated with psychotic experiences. The results are summarised in Table 3.

**Table 3:** Direct associations between nightmare symptoms, emotion dysregulation and psychotic experiences.

	*B*	*β*	95% CIs for *B*	Adjusted *R*^2^
*Step 1*				18%
Severity	−0.140	−0.046	−0.425 to 0.146	
Intensity	0.326	0.113*	0.063–0.589	
Awakenings	−0.249	−0.082*	−0.472 to −0.026	
Consequences	0.236	0.416***	0.191–0.280	
*Step 2*				32.4%
Severity	−0.168	−0.055	−0.430 to 0.093	
Intensity	0.320	0.111*	0.080–0.561	
Awakenings	−0.182	−0.060	−0.385 to 0.021	
Consequences	0.125	0.221***	0.080–0.170	
Strategies	0.101	0.100*	0.002–0.201	
Non-acceptance	0.089	0.090*	0.011–0.167	
Impulse	0.127	0.128**	0.047–0.207	
Goals	−0.020	−0.019	−0.102 to 0.062	
Awareness	0.091	0.077*	0.015–0.166	
Clarity	0.241	0.207***	0.155–0.326	

*Note*. *RLS/PLM* restless legs syndrome/periodic limb movement, *CRD* circadian rhythm disorder. *p < 0.05, **p < 0.005, ***p < 0.001.

### Indirect association between nightmare symptoms and psychotic experiences, via emotion dysregulation

**Fig. 1:**
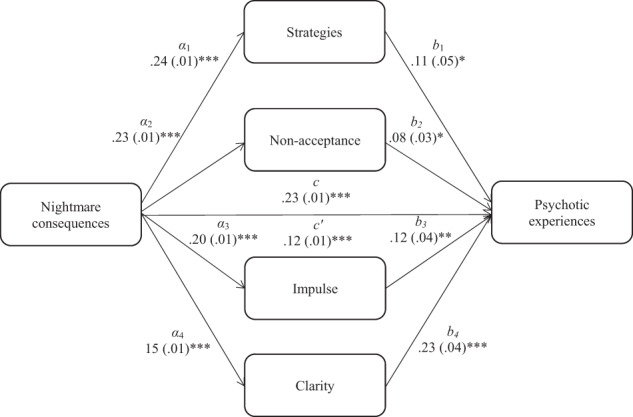
Indirect association between nightmare consequences and psychotic experiences. *Note*. The total (*c*) and the indirect effect (*c*′) of nightmare consequences on psychotic experiences are shown; unstandardised path coefficients are presented, with standard errors in brackets; *p < 0.05, **p < 0.005, ***p < 0.001. Strategies, limited access to adaptive emotion regulation skills; Nonacceptance, nonacceptance of emotional states; Impulse, difficulty controlling behaviours when upset; Clarity, lack of emotional clarity.

**Fig. 2:**
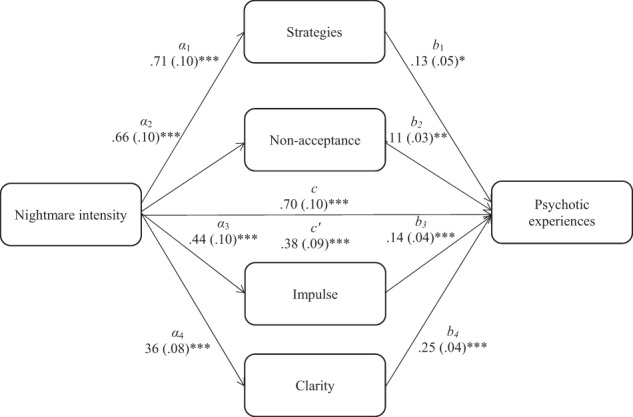
Indirect association between nightmare intensity and psychotic experiences. *Note*. The total (*c*) and the indirect effect (*c*′) of nightmare intensity on psychotic experiences are shown; unstandardised path coefficients are presented, with standard errors in brackets; *p < 0.05, **p < 0.005, ***p < 0.001. Strategies, limited access to adaptive emotion regulation skills; Nonacceptance, nonacceptance of emotional states; Impulse, difficulty controlling behaviours when upset; Clarity, lack of emotional clarity.

**Fig. 3:**
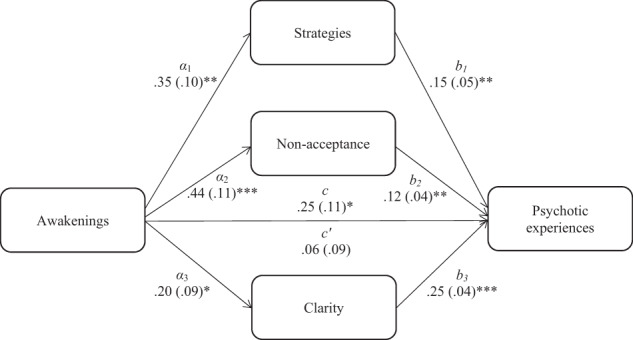
Indirect association between nightmare-induced awakenings and psychotic experiences. *Note*. The total (*c*) and the indirect effect (*c*′) of nightmare-induced awakenings on psychotic experiences are shown; unstandardised path coefficients are presented, with standard errors in brackets; *p < 0.05, **p < 0.005, ***p < 0.001. Strategies, limited access to adaptive emotion regulation skills; Nonacceptance, nonacceptance of emotional states; Impulse, difficulty controlling behaviours when upset; Clarity, lack of emotional clarity.

Regression-based multiple mediation modelling was used with the SPSS macro by Hayes^25^, in order to examine the indirect association between perceived nightmare consequences, intensity and resulting consequences, via the effects of the six emotion regulation difficulties (i.e., strategies, non-acceptance, impulse, goals, awareness and clarity). Three multiple mediation models were respectively examined for each predictor variable. Following Preacher and Hayes^26^ recommendations, bootstrapping with 1000 resamples and bias-corrected and accelerated confidence intervals were used, and the Sobel test (*z*) indicated the size of the mediation effect. The results from Model 1 (Fig. 1) showed that the association between perceived nightmare consequences and psychotic experiences was mediated by strategies (*z* = 2.26, *p* = 0.02), non-acceptance (*z* = 2.10, *p* = 0.03), impulse (*z* = 2.85, *p* < 0.005) and clarity (*z* = 4.69, *p* < 0.001). The results from Model 2 (Fig. 2) showed that the association between perceived nightmare intensity and psychotic experiences was mediated by strategies (*z* = 2.49, *p* = 0.01), non-acceptance (*z* = 2.61, *p* = 0.008), impulse (*z* = 2.70, *p* = 0.006) and clarity (*z* = 3.29, *p* = 0.001). Finally, the results from Model 3 (Fig. 3) showed that the association between nightmare-induced awakenings and psychotic experiences was mediated by strategies (*z* = 2.25, *p* = 0.02), non-acceptance (*z* = 2.49, *p* = 0.01) and clarity (*z* = 2.01, *p* = 0.04).

## Discussion

The primary purpose of this study was to evaluate the relationship between nightmare symptoms and psychotic experiences in university students, and also to examine the mediating role of emotion dysregulation. Our results showed that self-reported psychotic experiences were positively associated with different nightmare symptoms, although the observed effect sizes were small to moderate (*r* ~ 0.08–0.42). We, therefore, provide further evidence of increased reports of psychotic experiences among students presenting nightmare symptoms^8,9^. In line with previous work^9^ we moved away from the global assessment of nightmares which fail to decompose specific factors facilitating symptom severity scores. In our bivariate analysis, increased psychotic experiences were associated to the severity, intensity, frequency, resulting awakenings and perceived consequences of reported nightmares. More crucially, after accounting for shared variance among nightmare symptoms only the intensity, resulting awakenings and perceived consequences of nightmares were significantly associated with psychotic experiences. Together, these outcomes underscore the role of intensity and consequential distress resulting from the nightmare experience, rather than incidence, as key factors influencing psychotic symptoms among university students.

The experience of nightmare distress has previously been evidenced as more predictive of general psychiatric difficulty than nightmare frequency in students^9^. This outcome was confirmed among a small sample of patients reporting symptoms of psychosis, where nightmare distress was related to greater delusion severity, anxiety, stress, and depression^7^. Recently, students identified as high-risk for severe mental illness reported nightmares as more distressing relative to their low-risk counterparts^8^. These observations are important as they parallel examinations of positive symptoms of psychosis^7^ where distress accompanying the experience of voices and unusual beliefs differentiates the need for intervention^27–30^. While nightmare distress reliably indicates psychiatric difficulty and psychotic experiences^7–9^, the present outcomes shed light on mechanisms driving such distress in a non-clinical student population. In particular, psychotic experiences were predominately influenced by nightmare intensity, resulting awakenings and perceived consequences of nightmares which interfere with sleep quality, mood, mental and psychical health, and social and occupational functioning.

Difficulties in emotion regulation are considered key features of psychotic experiences^31^ and the production of nightmares^32,33^. While our results demonstrate the intensity, awakenings and consequences of nightmares to be associated with psychotic experiences, this outcome was differentially mediated by difficulties in emotion regulation. A surge in affective arousal (i.e. limbic activation, eye-movement, respiratory activity) is usually observed during rapid eye-movement sleep (REM)^14^. During the REM phase, it is speculated that adaptative regulation of dream content contain these surges through regulation of the intensity and variability of emotional content^14^^,^^34–37^. Generally, evidence points towards emotional experiences and thoughts prior to sleep as influencing the nature of dream content^32^^,33^. As dreams consequently influence mood state the following day^34^^,^^35^, persistently experiencing intense and distressing nightmares and associated consequences may indicate emotion regulation difficulties which may contribute to the development and maintenance of psychotic symptoms^7^^,^^14^. Indeed, a number of studies evidence emotion regulation difficulties in psychotic disorders. While outcomes remain mixed, this population reports difficulty in the identification of emotions^21^^,^^38–40^, and greater non-acceptance of their feelings^21^^,^^40,41^.

The DERS-SF examines the extent to which individuals present specific difficulties in emotion regulation that consequently interfere with social, cognitive or interpersonal functioning (i.e. non-acceptance, goals, impulse, awareness, strategies, clarity). In contrast, the commonly used Emotion Regulation Questionnaire^41–43^ presents a narrower focus by specifically exploring use of two strategies: cognitive reappraisal and expressive suppression. Antecedent in nature, reappraisal aims to modify the emotional meaning and impact of an emotional situation. Suppression, alternatively, is a response focused strategy that aims to actively inhibit emotional expression^24^. Previous examination of both measures found suppression to be associated with greater emotion regulation difficulty, whereas cognitive reappraisal was related to less difficulty^24^. In relation to the current outcomes, this may suggest reappraisal, relative to suppression, as favoured among those reporting psychotic experiences. However, additional research is required to confirm this notion.

The independent relationship between resulting awakenings and psychotic experiences should be taken with caution when considering the strength of the correlation coefficient (0.08). Here statistical significance may be explained by a Type 1 error resulting from the large size of the current sample. In contrast, this weak relationship could be the result of only measuring ‘resulting awakenings', which is one specific aspect of sleep disruption rather than an overall assessment of disturbed sleep in the context of nightmares. Several limitations of this work should be noted. First, no attention checks were used to exclude those who: experienced difficulty in concentration; or responded in a random manner. Moreover, while use of online self-report measures allowed for wider recruitment and consequential return of a large sample size, they are limited in depth and subjective accounts of sleep. That said, nightmare content, resulting distress and perception of functional impairment remain limited to subjective measures. While patient awakenings during/following polysomnographicly determined REM may gain novel patient insight regarding nightmare content, compromising sleep continuity and confounding examination nightmare-related awakenings would provide no additional insight in the context of the current examination. Next, by recruiting from multiple institutions from the UK, we expand on previous findings limited to a homogeneous sample of University of Oxford students^8^ potentially allowing a degree of generalisability to be made in the outcomes of both studies. However, the number of responses obtained from each institution were not recorded and may therefore indicate a sampling bias. Moreover, the cross-sectional design employed leaves the current outcomes vulnerable to inflation bias between variables and prevents the causality of the relationships identified from being conclusively defined. With that in mind, it is possible that the continual and disrupted experience of daytime distress associated with psychotic experiences influence the onset of nightmares^7^. Indeed, dreaming is considered to play a vital role in attenuating fear and regulation emotions^14^^,^^42,43^. That said, further research is required to clarify the causal direction of the relationship between the experience of nightmares and psychotic symptoms. Additionally, it is well established that females more frequently report nightmares and consequential distress, particularly during adolescence and young adulthood^44^. Given the current sample consisted mostly of female participants, this may limit generalisability to male students. Nevertheless, they still add valuable insight into relationship between nightmare symptoms and psychotic experiences. Finally, the DERS-SF allows the self-perception of emotion dysregulation to be explored on a large scale. While the subjective nature of this measure may present a limitation of the current work, it is relevant to note that: disturbing dreams, nightmares and psychotic experiences are largely determined through subjective report; patient perspectives allow specific treatment targets to be identified in talking therapies. That said, future work may consider taking a different approach to the assessment of emotional difficulties. Indeed, performance-based measures of emotional intelligence (i.e. perception, understanding, facilitation and management of emotion) such as the Mayer-Salovey-Caruso Emotional Intelligence Test^45^ may be considered.

Previous work highlights the importance of examining possible underlying mechanisms which may shed light on the relationship between the experience of nightmares and psychotic experiences. Here, we highlight the partially mediating role of specific impairments in emotion regulation. Additionally, for the first time we highlight the role of perceived psychical, psychological and interpersonal consequences of nightmares in predicting the extent of psychotic experiences in students. Relatedly, previous work highlights the influential role nightmare distress, rather than incidence, in determining psychological functioning. Together, these outcomes suggest nightmares and associated distress should be therapeutically targeted above existing cognitive behavioural treatment strategies^46^. Indeed, attenuation of paranoia and nightmare symptomology has recently been evidenced in a population experiencing persecutory delusions following a 4-week trial of image-focused cognitive behavioural therapy (CBT) for nightmares^46^.

Poor wellbeing and distress among university students is continually rising, with recent data highlighting a fivefold increase in the number of students revealing their mental health difficulties to institutional support services over the past decade^47,48^. Certainly, students face considerable life changes (e.g. increased independence, social demands) and academic challenges (e.g. independent learning) which may contribute to the experience of such psychiatric symptoms^47^. Therefore, appropriate screening of nightmare symptoms and emotion regulation difficulties among students reporting psychotic experiences may therefore guide student-support services when making judgments regarding treatment approach^47,48^. In particular, those presenting psychotic symptoms may benefit from a brief image-focused CBT intervention for nightmares^47^. Likewise, edifying appropriate use of emotion regulation strategies (e.g. guiding the patient to accept or actively decrease distress) may serve to increase the efficacy of such treatments in those experiencing emotional difficulties.

## Methods

### Sample and procedure

The study was approved by the Sheffield Hallam University Research Ethics Committee (Protocol number: ER7368595), and all participants provided online informed consent. As part of a larger project examining the prevalence of mental health difficulties among UK university students, a cross-sectional online questionnaire-based design was implemented. Students from six UK universities were recruited through institutional course participation schemes, social media groups and faculty emails. Specifically, Sheffield Hallam University, the University of Sheffield, Northumbria University, the University of Glasgow, Durham University and the University of York. This resulted in a sample of *N* = 1650 individuals who either began or clicked on a hyperlink to the survey which was delivered using the Qualtrics platform (Qualtrics, Provo, UT). Only complete cases were used in the analysis due to the ethical right to withdraw from the survey at any time. The data were also examined for duplicate responses based on matching IP addresses, where none were found. Therefore, *N* = 1273 respondents (mean age = 20.88 ± 4.53, range 18–56, 84% females) providing complete data (final response rate = 77.2%) for the variables of interest (i.e. psychotic experiences, nightmare disorder symptomology, emotion dysregulation) were entered into the final analysis. Students who requested course credit were remunerated on completion. SPSS (version 24, IBM Corp., Armonk, New York, United States) was used to perform formal statistical analyses of the data. See Table 4 for sample characteristics.

**Table 4:** Sample characteristics.

	Whole sample *N* = 1273	No nightmares indicated *N* = 516	Nightmares indicated *N* = 757
	*N*/mean ± SD	*N*/ mean ± SD	*N*/mean ± SD
Age (Mean ± SD)	20.88 ± 4.53	±	±
*Sex*			
Male	202	412	100
Female	1057	104	656
Non-binary	3	1	2
Gender-nonconforming	3	0	3
Unsure	5	1	3
Other	3	1	2
*Ethnic origin*			
White—United Kingdom	1024	395	629
White—Irish	15	7	8
White—Other	72	34	38
White and Black Caribbean	10	7	3
White and Black African	3	1	2
White and Asian	14	6	8
Mixed/multiple ethnic Other	14	7	7
Indian	28	13	15
Pakistani	17	9	8
Bangladeshi	6	3	3
Chinese	33	15	18
Asian Other	11	7	4
African	9	5	4
Caribbean	2	1	1
Black African/Caribbean Other	1	1	0
Arab	5	1	4
Other	9	4	5
*Course level*			
Undergraduate	1117	453	664
Postgraduate taught	79	27	52
Postgraduate research	17	8	9
Doctoral student	38	21	17
Postgraduate Other	22	7	15

±, standard deviation.

### Measures and materials

#### Psychotic experiences

The Prodromal Questionnaire 16 (PQ-16) was administered to assess life-time symptoms of psychotic experiences^49^. It was developed as a brief version of the 92-item Prodromal Questionnaire^50^ to enable the detection of ultra-high-risk (UHR) patients in routine adult mental health services. Sixteen items evaluate the occurrence of positive/negative symptoms and avolition on a two-point scale (yes/no). More specifically, the PQ-16 contains nine items relating to hallucinations; five items relating to delusions; and two negative symptom items. The summation of ‘yes’ item responses yield a total score between 0 and 16, where higher scores indicate an increased number of psychotic symptoms. In adults, a score of ≥6 predicts diagnosis of psychosis with high sensitivity (87%) and specificity (87%)^49^. The internal consistency (Cronbach’s alpha) of the scale in the current study was 0.85.

#### Disturbing dreams and nightmares

The Disturbing Dreams and Nightmare Severity Index (DDNSI) was used to examine nightmare complaints^51^. Seven items assess nightmare frequency (amount experienced per week); number of awakenings due to nightmares (0 = never/rarely, 4 = always); intensity of nightmares themselves (0 = not intense, 6 = extremely severe intensity); and the severity of the overall problem (0 = no problem, 6 = very severe problem). Total scores range between 0 and 37 with higher scores indicating greater difficulty with nightmares. The internal consistency of the scale in the present study was 0.75. The scale also presents an optional 10 item subscale which examines the extent of impairments which are perceived to be a consequence of the nightmare disturbance. Here, the summation of statements (e.g. my disturbing dreams or nightmares interfere with social or recreational activities: 0 = not at all, 3 = a great deal) yield a total score ranging between 0 and 30 with higher scores indicating greater perceived consequences of nightmares. Internal consistency of this subscale was 0.91.

#### Emotion dysregulation

The Short Form Difficulties in Emotion Regulation Scale (DERS-SF)^52^ assessed individuals’ ability to adequately regulate emotions. Six subscales assess difficulties in:

1. Non-acceptance: (non-acceptance of emotional states) items that reflect the tendency to experience negative secondary emotions in response to one’s negative emotions, or to have reactions of non-acceptance with respect to one’s own discomfort.
2. Goals: (difficulties engaging in goal directed behaviour in the context of emotional distress) items that reflect difficulty in concentrating on and pursuing a task when experiencing negative emotions.
3. Strategies: (limited access to adaptive emotion regulation skills) reflects a difficulty to effectively regulate the emotions that one has manifested.
4. Impulse: (difficulty controlling behaviours when upset) reflects difficulty in maintaining control of one’s behaviour when experiencing negative emotions.
5. Clarity: (lack of emotional clarity) includes items that reflect the degree to which one can understand distinctly what emotion one is experiencing.
6. Awareness: (lack of emotional awareness) contains items that emphasise the tendency to pay attention to emotions and the ability to recognise them.

Each subscale comprises three items scored on a 5-point likert scale ranging from 1 (almost never) to 5 (almost always). Mean scores are created for each subscale, with higher scores indicating greater emotion dysregulation. The DERS-SF is evidenced to capture aspects of emotion dysregulation measured by the original Difficulties in Emotion Regulation Scale (DERS)^53^, correlate with clinically relevant scales in a way that compares with those correlations observed when using the full DERS, and exhibits good internal reliability^52^. Internal consistency of subscales in the present study: Non-acceptance, 0.90; Goals, 0.92; Impulse, 0.93; Awareness, 0.82; Strategies, 0.90; and Clarity, 0.83.

### Reporting summary

Further information on research design is available in the Nature Research Reporting Summary linked to this article.

## Supplementary information

Reporting Summary

## Data Availability

The data that support the findings of this study are available from the corresponding author upon reasonable request.

## Code availability

The data codes that support the findings of this study are available from the corresponding author upon reasonable request.
